# Health Impact of Silver Nanoparticles: A Review of the Biodistribution and Toxicity Following Various Routes of Exposure

**DOI:** 10.3390/ijms21072375

**Published:** 2020-03-30

**Authors:** Zannatul Ferdous, Abderrahim Nemmar

**Affiliations:** 1 Department of Physiology, College of Medicine and Health Sciences, United Arab Emirates University, P.O. Box 17666 Al Ain, UAE; 201370004@uaeu.ac.ae; 2 Zayed Center for Health Sciences, United Arab Emirates University, P.O. Box 17666 Al Ain, UAE

**Keywords:** silver nanoparticles, cytotoxicity, routes of exposure, biodistribution

## Abstract

Engineered nanomaterials (ENMs) have gained huge importance in technological advancements over the past few years. Among the various ENMs, silver nanoparticles (AgNPs) have become one of the most explored nanotechnology-derived nanostructures and have been intensively investigated for their unique physicochemical properties. The widespread commercial and biomedical application of nanosilver include its use as a catalyst and an optical receptor in cosmetics, electronics and textile engineering, as a bactericidal agent, and in wound dressings, surgical instruments, and disinfectants. This, in turn, has increased the potential for interactions of AgNPs with terrestrial and aquatic environments, as well as potential exposure and toxicity to human health. In the present review, after giving an overview of ENMs, we discuss the current advances on the physiochemical properties of AgNPs with specific emphasis on biodistribution and both in vitro and in vivo toxicity following various routes of exposure. Most in vitro studies have demonstrated the size-, dose- and coating-dependent cellular uptake of AgNPs. Following NPs exposure, in vivo biodistribution studies have reported Ag accumulation and toxicity to local as well as distant organs. Though there has been an increase in the number of studies in this area, more investigations are required to understand the mechanisms of toxicity following various modes of exposure to AgNPs.

## 1. Introduction

### Nanotechnology

The concept of novel nanoscale technology was introduced in 1959 in a lecture of physicist Richard Feynman entitled “There is plenty of room at the bottom,” which discussed the importance of manipulating and controlling things at the atomic scale [1]. The term nanotechnology was first introduced by Professor Norio Taniguchi in 1974, and the American engineer Kim Eric Drexler popularized the concept of molecular nanotechnology by using it in his 1986 book Engines of Creation; The Coming Era of Nanotechnology [1]. However, it was only after the inventions of instruments for imaging surfaces at atomic level—the scanning tunneling microscope in 1981, which was followed by the atomic force microscope—that the growth of nanotechnology was sparked in the modern era [1]. The emerging and exponential nanotechnology involves the manipulation, design and precision placement of atoms and molecules at the nanoscale level [1]. Over the past two decades, nanotechnology has witnessed breakthroughs in the fields of medicine, environment, therapeutics, drug development, and biotechnology.

A major element of nanotechnology comprises engineered nanomaterials (ENMs). According to European commission, NMs are “a natural, incidental or manufactured material containing particles in an unbound state or as an aggregate or as an agglomerate and where, for 50% or more of the particles in the number size distribution, one or more external dimensions is in the size range of 1–100 nm” [2]. NMs-containing consumer products include cosmetics, electronics, kitchenware, textiles and sporting goods [3]. This wide utilization is due to unique properties of NMs, such as their small size, large surface area to volume ratio, high reactivity, high carrier capacity, and easy variation of surface properties [3]. The same unique properties that led to their widespread applications raise questions about potential environmental and health effects that might result from occupational exposures during the manufacture and use of NMs at the consumer end [4]. The toxic effect of NMs on humans has recently gained much attention in the health industry [5,6]. Most exposure to airborne NMs occurs in the workplace during the manufacture of these materials, the formulation of them into products, their transport, or their handling in the storage facilities [7]. Additionally, widespread consumer exposure via oral inhalation and direct contact with ENM-containing products is likely to occur [7]. Subsequently, the small size of this type of particle facilitates its translocation from natural barriers such as the gastrointestinal tract (GIT), lungs, or skin, and this translocation can induce acute and chronic toxic effects [8].

In spite of several advantages of nanoscale materials, their potential health hazards cannot be overlooked due to their uncontrollable use, discharge to the natural environment and potential toxic effects. Hence, nanotoxicology warrants intensive research studies in make the use of NMs more convenient and environment friendly. Some of the most commonly studied NMs include fullerenes, carbon nanotubes (CNTs), silver nanoparticles (AgNPs), gold nanoparticles (AuNPs), titanium oxide nanoparticles (TiO_2_), zinc oxide nanoparticles, iron oxide (FeO), and silica nanoparticles [9]. Among these, AgNPs have gained strong popularity among researchers over the past few decades [9]. Initial investigations on AgNPs were more focused on synthesizing and characterizing them by using chemical approaches [10]. However, current works have been more concentrated on their biological effects and applications for several purposes [11]. AgNPs are also known to have unique properties in terms of toxicity, surface plasmon resonance, and electrical resistance [11]. Based on these, intensive works have been conducted to investigate their properties and potential applications for several purposes such as antimicrobial agents in wound dressings, water disinfectants, electronic devices, and anticancer agents [11,12].

Previous review papers have addressed the toxicological properties of AgNPs during their use as antimicrobial agents for textiles, dental biomaterials, and bio-detectors, as well as during their syntheses [13,14,15,16,17,18]. Recent reviews have covered topics such as their biosynthesis by using plant extracts for antimicrobial applications, biocidal properties, and cytotoxicity based on their physiochemical properties such as size, concentration and coating [19,20,21]. Moreover, the chemical and toxicological interactions of AgNPs with other metal NPs such as Ag, Fe, and TiO_2_ were also discussed by Sharma et al. [22]. An important factor regarding the potential health impact of AgNPs is their various routes of exposure. In this regard, the potential toxic effects of AgNPs after oral exposure were also thoroughly discussed by Gaillet et al. [23]. However, other major routes including respiratory, dermal and intravenous exposure have not been covered in previous review articles. In our present paper, we summarize the existing physical, chemical, and biological synthetic approaches of AgNPs, followed by a description on their characterization techniques and applications. Notably, our paper aims to report the most recent update and important studies on the toxic effect of AgNPs following various routes of exposure including oral, inhalation, dermal, and intravenous administration. Moreover, important in vitro and in vivo pathophysiological effects at the site of exposure and in remote and distant organs following exposure are highlighted. To our knowledge, no other previous reviews have structurally presented these topics so far. We conducted an extensive literature search of bibliographic databases (e.g., PubMed, Google Scholar, Medline, and Web of Science) by using different keywords and combinations of keywords (AgNPs, biodistribution, in vitro toxicity, in vivo toxicity, pulmonary exposure, inhalation, instillation, oral exposure, dermal exposure and organ toxicity) to retrieve the relevant information.

## 2. Silver Nanoparticles (AgNPs)

Of all the ENMs developed and characterized so far, AgNPs has assumed a significant position due to their potential uses in commercial applications [12,24]. Silver, symbolized as Ag, is a lustrous, soft, ductile and malleable metal that has the highest electrical conductivity of all metals and is widely used in electrical appliances [25]. This precious metal is chemically inactive, stable in water, and does not oxidize in air—hence, it is used in the manufacturing of coins, ornaments and jewelry [25]. Silver can be obtained from pure deposits as well as from silver ores such as horn silver and argentite. Most silver is derived as a by-product along with deposits of ores containing gold, copper, and lead [25]. It is estimated that nearly 320 tons of nanoparticulate form of Ag are manufactured every year and used in nanomedical imaging, biosensing and food products [21]. AgNPs exhibit special properties relative to their bulk components due to their unique physicochemical properties including their small size, greater surface area, surface chemistry, shape, particle morphology, particle composition, coating/capping, agglomeration, rate of particle dissolution, particle reactivity in solution, efficiency of ion release, and type of reducing agents used for the synthesis of AgNPs [12,26]. In addition, AgNPs are also well known for their antimicrobial, optical, electrical, and catalytic properties [11]. Owing to their unique properties, AgNPs have been extensively used in household utensils, food storage, the health care industry, environmental applications, and biomedical applications such as wound dressings, surgical instruments, and disinfectants [26]. Furthermore, due to their optical activities, these NPs have been used in catalysis, electronics and biosensors [26].

### 2.1. AgNPs Synthesis and Characterization

Numerous methods have been adopted for the synthesis of AgNPs in order to meet these increasing requirements. The conventional physical method of synthesis includes spark discharge and pyrolysis [27,28]. A chemical method that can be a top–down or bottom–up approach involves three main components: metal precursors, reducing agents and stabilizing/capping agents [12]. The common approach is usually chemical reduction by organic or inorganic reducing agents, such as sodium citrate, ascorbate, sodium borohydride, elemental hydrogen, polyol process, Tollens reagent, N, N-dimethylformamide, and polyethylene glycol-block copolymer [12]. Other procedures include cryochemical synthesis, laser ablation, lithography, electrochemical reduction, laser irradiation, sono-decomposition and thermal decomposition [12]. The major advantage of the chemical method is its high yield unlike the physical method, which has a low yield [10]. However, contrary to the physical method, the chemical method is extremely expensive, toxic and hazardous [10]. In order to overcome the later limitations, the biologically-mediated synthesis of NPs has emerged as a better option. This simple, cost effective and environment friendly approach uses biological systems including bacteria, fungi, plant extracts, and small biomolecules like vitamins, amino acids and enzymes for the synthesis of AgNPs [29]. The green approach is widely accepted due to the availability of a vast array of biological resources, a decreased time requirement, high density, stability, and the ready solubility of prepared NPs in water [29].

The characterization of AgNPs is a very crucial step to evaluate the functional effect of synthesized particles [12]. It has been documented in various studies that the biological activity of AgNPs depends on morphology, structure, size, shape, charge and coating/capping, chemical composition, redox potential, particle dissolution, ion release, and degree of aggregation [21,30,31,32,33]. Like all other NPs, these parameters can be determined by using various analytical techniques, such as dynamic light scattering (DLS), zeta potential, and advanced microscopic techniques such as atomic force microscopy, scanning electron microscopy (SEM) and transmission electron microscopy (TEM), UV-vis spectroscopy, X-ray diffractometry (XRD), Fourier transform infrared spectroscopy (FTIR), and X-ray photoelectron spectroscopy (XPS) [12,34]. To capture the concept of importance of AgNPs characterization, it must be noted that from a toxicological perspective, studies that used similar AgNPs provided by the same manufacturer demonstrated different results. For example, a repeated exposure study by Vandebriel RJ et al. [35] in rats showed that AgNPs are cytotoxic to various cells. On the other hand, Boudreau M.D et al. [36], using similar particles, showed the dose-dependent accumulation of AgNPs in various tissues of rats, without causing significant cytotoxicity. Moreover, some published nanotoxicity studies have reported characteristics of the particles by using only manufacturer’s data without investigators to confirm their found characteristics or by using single analytical tool that provides limited information about the particle-type being studied [35,37,38,39]. This brings in the importance of the adequate physicochemical characterization of AgNPs prior to undertaking toxicity assessment studies. In addition, a standardized measurement approach like the application of validated methods and the use of reference materials that are specific to AgNPs needs to be more developed in order to assure the comparability of results among toxicity studies that used similar AgNPs.

### 2.2. AgNPs Physicochemical Properties

Size is an important property that influence the NPs uptake and effect. In this regard, a review study reported that the common sizes of AgNPs used in general applications ranging from 1 to 10 nm [40]. The reason behind this is that smaller particles have been found to display better antimicrobial and cytotoxic activity, as demonstrated in various studies [38,41,42]. Furthermore, as the particle size gets smaller, the specific surface area increases and, hence, a greater proportion of its atoms is displayed on the surface [4] This implies that for the same mass of an NP, biological interactions and toxicity are more dependent on particle number and surface area than on particle mass. The properties of AgNPs can be further enhanced by the functionalization of NPs by using various coatings that in turn influence NPs’ surface charge, solubility, and/or hydrophobicity. There is considerable literature that has suggested that the fate and toxicity of AgNPs are determined by their types of coating [43,44]. Various types of surface coatings applied to AgNPs, particularly to improve their biocompatibility and stability against agglomeration are trisodium citrate (CT-AgNP), sodium bis(2-ethylhexyl) sulfosuccinate, cetyltrimethylammonium bromide, polyvinylpyrrolidone (PVP-AgNP), poly(L-lysine), bovine serum albumin, Brij 35 and Tween 20 [45]. Among the latter, CT and PVP coatings are the most commonly used as stabilizing agents [40]. Furthermore, the coating also modifies their charge, which again influences their toxic effect in cells. For example, positively charged NPs are considered more suitable drug delivery tools for anticancer drugs because they can stay for a long time in the blood stream compared to negatively charged particles [12]. Shape-dependent effects were also reported in studies by using varying sized AgNPs. For instance, silver nanocubes showed greater antibacterial effect against *Escherichia coli* compared to spheres and wires in a study that used 55 nm AgNPs [46]. Pal. S et al. [47] also successfully demonstrated a shape-dependent interaction with *E. coli* where truncated a triangular-shaped AgNP had stronger biocidal action than spherical and rod shaped AgNPs. Contrarily, Actis et al. [48] reported no biocidal effect on *Staphylococcus aureus* after using spherical, triangular and cuboid AgNPs. Cellular uptake and biological responses are also defined by the agglomeration state of NPs, and there is sufficient evidence that interaction of the AgNPs with biological media and biomolecules can lead to particle agglomeration and aggregation [49,50]. Though the easy penetration of agglomerated AgNPs in mesenchymal stems cells and nuclei have been reported in several studies, reduced cytotoxicity has also been evident with agglomerated particles compared to free AgNPs [49,51]. A good amount of research has also been conducted on various types of surface corona resulting from interfacial interactions between AgNPs and biological fluids [20]. This has included studies involving both single and complex molecule protein coronas like bovine and human serum albumin, tubulin, ubiquitin, and fetal bovine serum [52]. The formation of a corona, depending on composition, has been shown to interfere with AgNPs’ dissolution to Ag ions and, thus, their toxicity [52]. Researchers have also successfully established the importance of various AgNP formulation during synthesis with respect to biomedical applications [53]. For example, the loading of AgNPs inside multiwalled carbon nanotubes has demonstrated an improved targeting of AgNPs to sperm cells and, hence, the potential for development as diagnostic tools for infertility management [54]. Similarly, Bilal et al. [55] synthesized an AgNPs-loaded chitosan-alginate construct that interestingly showed excellent biocompatibility with normal cell line (L929) and cytotoxicity to cancer cells (HeLa cells). Azizi et al. [56] formulated albumin-coated AgNPs with the aim of developing new anticancer agents and showed that the latter was taken specifically by tumor cells and induced apoptosis.

### 2.3. AgNPs Application and Mechanism of Action

Among various metal salts and NMs that are known to be effective in inhibiting the growth of many bacteria, AgNPs are noteworthy for their strong inhibitory and bactericidal effects [57,58]. The use of AgNPs as well as Ag salts in catheters, cuts, burns and wounds to protect them against infection has been well established [59,60,61,62]. However, the exact mechanism underlying the antimicrobial effects of AgNPs is still unresolved, though the literature has suggested that these particles can interact with the membranes of bacteria [15,63]. A potential proposed pathway is that AgNPs, upon interaction with bacteria, induce reactive oxygen species and free radicals, thus damaging the intracellular organelles and modulating the intracellular signaling pathways towards apoptosis [64]. Another widely accepted mechanism of bacterial cytotoxicity is the adhesion of AgNPs to the bacterial wall, followed by the infiltration of the particles, with bacterial cell membrane damage leading to the leakage of cellular contents and death [63,65]. In this context, the antimicrobial activity assessment of small sized AgNPs (12 nm) by Das et al. [66] demonstrated these NPs to be excellent inhibitors against both Gram-positive and Gram-negative bacteria, including *Staphylococcus bacillus*, *Staphylococcus aureus*, and *Pseudomonas aeruginosa*. This indicates that both the membrane thickness and surface charge facilitates particle attachment onto the cell membrane [67]. Finally, the large surface area of AgNPs releasing Ag^+^ ions is another crucial factor that contributes to the cytotoxic activity. As it is well established, smaller AgNPs have a faster rate of silver ion (Ag^+^) dissolution in the surrounding microenvironment due to their larger surface area to volume ratio and, hence, an increased bioavailability, enhanced distribution, and toxicity of Ag compared with larger NPs [68,69]. Furthermore, Ag^+^ ions’ release rate is dependent on a number of factors including the size, shape, concentration, capping agent and colloidal state of NPs [70,71]. In particular, the rate of Ag^+^ ion release has been shown to be associated with the presence of chlorine, thiols, sulfur, and oxygen [14]. Released Ag^+^ ions are suggested to interact with respiratory chain proteins on the membrane, interrupt intracellular O_2_ reduction, and induce reactive oxygen species (ROS) production, thus causing cellular oxidative stress in microbes and death [72]. AgNPs are also familiar for their antifungal, antiviral and anti-inflammatory activity [71]. Several studies have reported the potent anti-fungal activity of AgNPs against several phytopathogenic fungi (e.g., *Alternaria alternate*, *Sclerotinia sclerotiorum*, *Macrophomina phaseolina*, *Rhizoctonia solani*, *Botrytis cinereal* and *Curvularia lunata)* as well as human pathogenic fungi (e.g., *Candida* and *Trichoderma sp*) [73,74]. Likewise, AgNPs have demonstrated efficient inhibitory activities against several viruses including human immunodeficiency virus, hepatitis B virus, herpes simplex virus, human parainfluenza virus, peste des petits ruminants virus, and bean yellow mosaic virus, a plant pathogenic virus [75,76,77,78,79]. Inflammation is an early immunological response against foreign particles by tissue, and, interestingly, AgNPs have been recently recognized to play important roles as anti-inflammatory agents. Studies evaluating the anti-inflammatory effect of AgNPs have shown a significant reduction in wound inflammation, a modulation of fibrogenic cytokines, a down regulation of pro-inflammatory cytokines, and apoptosis in inflammatory cells [61,80,81].

## 3. Routes of Exposure and Biodistribution

**Figure 1:**
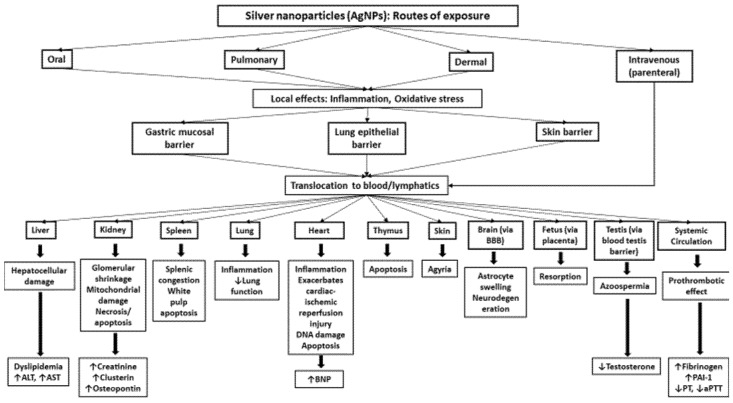
Schematic representation of the biodistribution and toxicity of silver nanoparticles (AgNPs) following various routes of exposure.

The major routes of entry of NPs are ingestion, inhalation, dermal contact and, directly in systemic circulation via intraperitoneal (i.p.) or intravenous (i.v.) injection [7]. The various modes of exposure to AgNPs, their biodistribution, and their mechanisms underlying the effects are illustrated in Figure 1. As AgNPs are extensively used in various household and biomedical products, the following section discusses the various potential routes of entry of these NPs.

After their exposure, AgNPs are able to induce inflammation and oxidative stress at the site of exposure. Moreover, they can cross various biological barriers and enter the systemic circulation. Intravenously-administered AgNPs are directly available in circulation. From then onwards, AgNPs are distributed to various organs and cause organ-specific pathophysiological effects. It remains to be seen whether the effects observed in the distant organs are due to the direct impact of the translocated AgNPs and/or particle-induced inflammatory and oxidative stress responses at the site of exposure. Some abbreviation are as follows: alanine aminotransferase (ALT), aspartate aminotransferase (AST), brain natriuretic peptide, plasminogen activator inhibitor-1, prothrombin time, activated partial thromboplastin time, blood–brain barrier.

### 3.1. Respiratory Exposure

The release of AgNPs in the environment during the manufacturing, washing or disposal of products enables the NPs to enter the human respiratory system through inhalation [82]. An exposure assessment in an NMs manufacturing facility showed a significant release of AgNPs during processing as soon as the reactor, dryer and grinder were opened, leading to potential occupational exposure even for wet production processes [83]. Similar studies evaluating workplace exposure and health hazard have reported that concentrations of AgNPs in the processes of manufacturing and integration of AgNPs into various consumer products can reach up to 1.35 μg/m^3^ [84,85]. Several healthcare, hygiene and antibacterial spray products containing AgNPs have now entered in our daily use. Nazarenko et al. [86] and Lorenz et al. [87] reported that the use of nanotechnology-based consumer sprays containing AgNPs can lead to the generation of nanosized aerosols and the release of NPs near the human breathing zone. Furthermore, Ag-treated textiles can be a source of AgNPs in washing solutions when laundering fabrics, regardless of either conventional Ag or nano Ag treatment [88]. A recent study that evaluated the effluent from a commercially available silver nanowashing machine showed that AgNPs, at an average concentration of 11 µg/L, were released in the environment [89]. AgNPs with a maximum concentration of 145 µg/L were also reported to be released from the outdoor paints during initial runoff events [90]. An occupational study in a silver manufacturing plant revealed that AgNPs in the air increases during production, and a peak area concentration of more than 290 µg/m^3^ could be been detected [84]. The authors suggested the possibility for workers to be exposed to airborne AgNPs at concentrations ranging from 0.005 to 0.289 mg/m^3^. In spite of considerable studies that have evaluated pulmonary exposure and toxicity, there are still a lack of long-term toxicity data, consumer exposure data, and human health effect data on AgNPs information. Nevertheless, an occupational exposure limit of 0.19 μg/m^3^ for AgNPs has recently been proposed based on a subchronic rat inhalation toxicity study and by taking the human equivalent concentration with kinetics into consideration [91]. Following inhalation, the transport and deposition of NPs is not uniform and is influenced by several factors including flow rate, the structure of the airway, pulmonary function, age, and, most importantly, particle size [82]. Particles smaller than 0.1 μm have been shown to penetrate deeply into the alveolar region, mainly by diffusion [7,92]. Consequently, due to deeper particle deposition, the clearance mechanism takes longer and leads to prolonged particle–tissue interactions and more pathophysiological effects [7]. In addition, translocation in blood capillaries is relatively easy for particles with a diameter lower than 0.1 μm [7]. The alveolar–capillary barrier consists of a very thin monolayer of epithelial cells, the endothelial cells of the capillaries, and the basement membrane between the two cells, and this barrier maintains the homeostasis of the lung [7]. NP penetration has been demonstrated following damage to the epithelial layer of the alveolar capillary membrane [7,93].

### 3.2. Oral Exposure

In the food industry, AgNPs are used in packaging and storage in order to increase the shelf life and quality of food [23]. Moreover, urban and industrial effluents enter the aquatic ecosystem and accumulate along trophic chains [94]. Thus, the presence of AgNPs in dietary supplements, water contamination, or food fish and other aquatic organisms provides the potential sources of oral exposure [23]. Recent studies have also demonstrated that AgNPs incorporated in food packaging can migrate from packaging into food under several usage conditions [95,96]. Inhalation exposure during manufacturing also ultimately leads to oral exposure, since particles cleared via the mucociliary escalator are swallowed and cleared through the GIT. It is estimated that the amount of daily consumption of silver in humans by ingestion is around 20–80 µg [7]. After ingestion, the GIT serves as a mucosal barrier that selectively promotes the degradation and uptake of nutrients such as carbohydrates, peptides, and fats. NPs can act on the mucus layer, translocate to the blood stream and consequently access each organ upon crossing the epithelium. It has been reported that the uptake of NPs with a diameter lower than 100 nm occurs mainly by endocytosis in epithelial cells [97]. Within enterocytes, AgNPs can trigger oxidative stress, DNA damage, and inflammation [7].

### 3.3. Skin and Parenteral Exposure

Human exposure to AgNPs may also take place through the skin, the largest organ of the body and the first line of defense between the external environment and the internal environment. The potential of solid NPs to penetrate healthy and breached human skin, as well as their ability to diffuse into underlying structures, has been well demonstrated [98,99]. In this context, the use of AgNPs in cosmetics production has been estimated to reach up to 20%. In addition to cosmetics, dermal contact to wound dressings and antibacterial textiles has also shown large diffusion of AgNPs [100]. In a laboratory set up, i.v., i.p., and subcutaneous injection enables AgNPs to directly gain access into systemic circulation. Furthermore, the development of AgNP-based drugs or drug carriers could enable the direct entry of these particles into the human circulatory system.

Following exposure, the distribution and toxicity of AgNPs is further discussed broadly under the in vivo toxicity section of this review article. In general, exposure and gender-related differences in the target tissue AgNP accumulation have been evident in previous research [101,102,103,104]. Next to biodistribution, the assessment of the clearance behavior of NPs is an important indicator of cumulative toxicity. In this context, there are several studies that have investigated the post exposure clearance kinetics following subacute inhalation, i.v, and, oral exposure to various sizes of AgNPs and Ag^+^ ions [104,105,106,107]. These studies have revealed silver clearance from most organs after the recovery period, which is generally 17 days to four months. However, tissues with biological barriers like the brain and testes have exhibited a persistence of silver in long term oral exposure studies, suggesting the difficulty of the silver to be cleared from these organs [106,107]. The persistence of silver in these organs also enhances chances of increased toxicity.

## 4. Pathophysiological Effects of AgNPs

The increasing concern about the possible impact of AgNPs on the environment and human health has directed researchers to focus on the in vitro and in vivo toxicity induced by these particles.

### 4.1. In Vitro Effects

In vitro cytotoxicity studies are often used to characterize the biological response to AgNPs, and the results of these studies may be used to identify hazards associated with exposure to AgNPs. Some important studies that have shown the toxic effects of AgNPs on different cell lines, including macrophages (RAW 264.7), including bronchial epithelial cells (BEAS-2B), alveolar epithelial cells (A549), hepatocytes (C3A, HepG2), colon cells (Caco2), skin keratinocytes (HaCaT), human epidermal keratinocytes (HEKs), erythrocytes, neuroblastoma cells, embryonic kidney cells (HEK293T), porcine kidney cells (Pk 15), monocytic cells (THP-1), and stem cells [20,108,109,110,111,112,113,114,115,116,117], are discussed below.

The exposure of A549 cells to increasing concentrations of AgNPs for 24 h has been to shown cause morphological changes including cell shrinkage, few cellular extensions, a restricted spreading pattern, and cell death in a dose-dependent manner [118]. In another study that used the same type of cells, treatment with 20 nm AgNPs induced DNA damage and the overexpression of metallothioneins at a concentration of 0.6 nM up to 48 h [119]. Size-dependent changes in cellular morphology were observed in a rat alveolar macrophage cell line incubated with hydrocarbon-coated AgNPs of different sizes (15, 30 and 55 nm) [120]. Gliga et al. [31] explored the mechanism of toxicity in BEAS-2B cells exposed to CT-AgNPs of different particle sizes (10, 40 and 75 nm) as well as to 10 nm PVP-coated and 50 nm uncoated AgNPs. In the latter study, cytotoxicity was evaluated with Alamar Blue and lactate dehydrogenase (LDH) assay. The Alamar Blue reagent assessed cell viability and proliferation based on the reduction potential of metabolically active cells. Their results showed cytotoxicity only of the 10 nm particles, independently of surface coating, and toxicity observed was associated with the rate of intracellular Ag release, a ‘Trojan Horse’ effect. Nguyen et al. [44] exposed a macrophage cell line to uncoated (20, 40, 60, and 80 nm) and PVP-AgNPs (10, 50, and 75 nm) and found a cell shrinkage effect due to uncoated particles, whereas cell elongation was evident after treatment with PVP-coated particles. The exposure of BEAS-2B and RAW 264.7 cell lines to 20 and 110 nm PVP- and CT-Ag-AuNPs (AgNPs with a gold core) showed that 20 nm Ag-AuNPs induced a significant reduction in cell viability in the dose range of 6.25–50 μg/mL for 24 h [121]. In addition, significant ROS generation, intracellular calcium influx, and a decline in mitochondrial membrane potential were also demonstrated in 20 nm CT- and PVP-AgNPs and 110 nm CT-AgNP-treated cells. Bastos V. et al. [122] also evaluated the cytotoxicity of 30 nm CT-AgNPs on RAW 264.7 cells by using parameters including viability, oxidative stress, and cytostaticity at 24 and 48 h of exposure. Their findings revealed decreased cell proliferation and viability at a concentration of only 75 μg/mL, thereby suggesting the low sensitivity of RAW 264.7 cells to lower doses of AgNPs. Recently, Gliga et al. [116], using a combination of RNA sequence and functional assays, showed that repeated, low doses (1 µg/mL) and long term exposure (six weeks) of BEAS-2B cells to 10 and 75 nm CT-AgNPs is profibrotic, indicated by the upregulation of *TGFβ1* and induce epithelial–mesenchymal transition and cell transformation. This evidence suggests that the observed cellular effects are dose-, size-, coating- and duration of exposure-dependent.

The exposure of 20 nm AgNPs to C3A cells at sublethal concentrations (1.95 µg/10^6^ cells) revealed size-dependent cytotoxicity, as indicated by elevated LDH levels, an increased release of inflammatory proteins (interleukin (IL) 8 and tumor necrosis factor (TNF) α), oxidative stress, and a decrease in albumin synthesis [123]. Cell viability was also evaluated by a 3-(4,5-dimethylthiazol-2-yl)-2,5-diphenyl tetrazolium bromide (MTT) assay, a colorimetric assay measuring cell metabolic activity based on nicotinamide adenine dinucleotide phosphate -dependent cellular oxidoreductase enzymes, in human hepatoblastoma HepG2 and mice primary liver cells. Interestingly, AgNPs caused a concentration-dependent decrease of cell viability in both cell types [124]. A study by Xue et al. [125] in HepG2 cells demonstrated that AgNPs are able to cause time- (24 and 48 h) and dose-dependent (40, 80, 160 µg/mL) decreases in cell viability, and they are induce cell-cycle arrest in the gap/mitotic phase, significantly increasing the apoptosis rate and ROS generation. A similar study that used PVP and CT-AgNPs at concentrations of 1–100 mg/L also showed coating- (with CT causing more effects than PVP) and dose-dependent reductions in cell viability along with the inhibition of albumin synthesis, as well as a decrease in alanine transaminase activity and apoptosis in HepG2 cells, thus indicating the therapeutic potential of the AgNPs against hepatic cancer [126]. Intestinal cells treated with Ag showed an induction of cytokine release and a higher genotoxicity compared to other inorganic metallic NPs (TiO2 and silicon dioxide) [127]. Böhmert et al. [128] analyzed the toxicity of AgNPs with a primary size of 7.02 ± 0.68 nm in Caco-2 cells by using NP concentrations between 1 and 100 μg/ml. A partial aggregation between digested and not-digested particles was observed by field fractionation (A4F) combined with DLS and X-ray dispersion at small angles. The authors concluded that AgNPs entered the GIT barrier without forming large aggregates in digestive fluids. These results confirmed the importance of body fluids on NP behavior and toxicity.

Samberg et al. [129] assayed the potential cytotoxicity of AgNPs in HEKs cells following 24 h of exposure and reported that unwashed and uncoated AgNPs caused a significant dose-dependent decrease of HEK cell viability and an increase in inflammatory cytokines, whereas washed and carbon-coated AgNPs did not induce any effect. Moreover, an in vitro percutaneous penetration of Ag study revealed that the accumulation of Ag and silver chloride aggregates of smaller than 1 µm, both in the epidermis and dermis [130].

NPs readily enter systemic circulation and may interact with circulatory components like blood cells, the heart, and blood vessels [131,132,133]. The potential impacts of human exposure to AgNPs on hemolysis, platelet activity and coagulation have recently gained interest. Studies that used human erythrocytes have investigated the effects of AgNPs on hemolysis, morphology, and their uptake [132,134]. In our recent in vitro study, we assessed the effect of the coating and dose of AgNPs on oxidative damage and eryptosis on mice erythrocytes [111]. Both PVP and CT-AgNPs induced oxidative stress and increased cytosolic calcium, annexin V binding, and calpain activity. The latter data may explain the mechanism of hemolysis and eryptosis induced by AgNPs. These NPs could also prevent platelet responses, as evidenced by the inhibitory effects of AgNPs of different sizes (13–15 , 30–35,  and 40–45 nm) on platelet aggregation [135]. Conversely, Bian et al. [136] recently compared the AgNPs (<100 nm) with Ag macro particles (5–8 µm) and showed that the former can promote phosphatidylserine (PS) exposure and microvesicle generation in freshly isolated human erythrocytes, mainly through ROS generation and intracellular calcium increases, hence suggesting that AgNPs may have prothrombotic risks by promoting the procoagulant activity of red blood cells, more importantly at non-hemolytic concentrations (≤100 μg/mL). These discrepancies in results, though not fully understood, could be related to the different cell types used in studies. The various blood biological effects of AgNPs, such as hemolysis, the interference of plasma coagulation, the enhancement of platelet aggregation, and the inhibition of lymphocyte proliferation, are size-, coating- and concentration-dependent [111,137,138]. Lin et al. [139] studied the potential toxicity of AgNPs on cardiac electrophysiology. The particles caused the concentration-dependent (10^−9^–10^−6^ g/mL) depolarization of resting membrane potential and diminished action potential, subsequently leading to a loss of excitability in mice cardiac papillary muscle cells in vitro. Milic M et al. [113] investigated the interaction of CT-AgNPs (13–61 nm) with porcine kidney (Pk15) cells, and compared the effect of the particles in their ionic form. For both forms of silver, concentration (1–75 mg/L) dependently decreased the viability of Pk15 cells after 24 h.

Furthermore, AgNPs exhibited an increased toxicity in stem cells, and this was attributed to their properties such as size, concentration and coating. The biocompatibility of 100 nm AgNPs was tested in human mesenchymal stem cells (hMSCs) by Greulich C et al. [140], and there was a dose-dependent (0.5–50 µg/mL) effect on cytotoxicity exhibited by decreased cell proliferation and chemotaxis. In addition, He W et al. [141] showed an increased LDH release and ROS production and reduction in both cell viability and mitochondrial membrane potential in hMSCs exposed to 30 nm AgNPs. Murine spermatogonial stem cells showed less cell viability, LDH leakage, and prolonged apoptosis after exposure to 15 nm AgNPs at concentrations of 5, 10, 25, 50, and 100 μg/mL [142]. Similarly, neural stem cells (NSCs) showed an increase in cell death and LDH leakage, an induction of ROS, an upregulation of the pro-apoptotic Bax protein, and increased in apoptosis when exposed to various concentrations (0.01–80 μg/mL) of PVP-Ag-NPs [143]. In the latter study, AgNPs induced neurotoxicity was compared to Ag^+^ ions, and the authors demonstrated that AgNPs caused cell apoptosis by inducing intracellular ROS generation coupled with c-Jun N-terminal kinases phosphorylation, while Ag ions caused cell necrosis via the alteration of cell membrane integrity and direct binding with cellular thiol groups.

Results of in vitro studies have indicated that AgNPs are toxic to the mammalian cells that are derived from the skin, the liver, the lung, the brain, the vascular system and reproductive organs [144]. The cytotoxicity of AgNPs depends on their size, shape, surface charge, coating/capping agent, dosage, oxidation state, agglomeration and type of pathogens against which their toxicity is investigated [42,108,145,146]. Despite these studies, the toxicological of AgNPs mechanism is still unclear. Several studies have reported DNA damage and apoptosis induced by NPs. In this context, AgNPs have been shown to cause apoptosis in mouse embryonic stem cells [147]. A follow up by the same group also demonstrated the involvement of AgNPs in the activation of apoptotic markers, caspase 3 and caspase 9, at concentrations of 50 and 100 µg/mL [148]. DNA damage at a concentration of 0.1 µg/mL of AgNPs was also reported in a study that investigated chromosomal aberrations in human mesenchymal cells [149]. Furthermore, the potential of AgNPs to induce genes that are associated with cell cycle progression, cause chromosomal damage, cell cycle arrest, and cell death in human BEAS-2B cells, umbilical vein endothelial cells, and hepatocellular liver carcinoma cells at various concentrations was also reported [144]. In spite of these numerous studies, a major limitation of the in vitro study of hazard identification with respect to human health is related to the doses used in in vitro studies, as these doses may not be comparable to realistic exposure doses in human. Hence, this necessitates in vivo toxicity research, a review of which is presented in the following section based on potential routes of exposure.

### 4.2. In Vivo Toxicity

In vivo biodistribution and toxicity studies in rats and mice have demonstrated that AgNPs that are administered by inhalation, ingestion or i.v./i.p. injection are subsequently detected in blood and cause toxicity in several organs including the lung, the liver, the kidney, the intestine and the brain.

Inhalation is a proposed major route of exposure, not only during manufacturing of Ag-containing materials but also during the use of aerosolized products. Table 1 and Table 2 summarize the important toxicity and biodistribution studies of AgNPs in rodents following pulmonary exposure via inhalation and intratracheal (i.t.) instillation, respectively. The data from these studies showed diverse outcomes related to biodistribution and remote organ toxicity. Some studies showed no induction of adverse effects [150,151], while other studies reported adverse effects varying from a minimal inflammatory response to the presence of inflammatory lesions in the lungs [103,104,152,153]. For instance, a 28-day inhalation toxicity study on rats showed no significant changes in the hematology and blood biochemistry in either the male or female rats following exposure to 11–14 nm AgNPs at concentrations of 1.73 × 10^4^/cm^3^, 1.27 × 10^5^/cm^3^ and 1.32 × 10^6^ particles/cm^3^ [151]. Hyun et al. [154] also exposed rats to 12–15 nm AgNPs for similar durations and doses and showed no remarkable histopathological changes in the nasal cavity and the lung in the NPs exposed group compared to the control group. Nevertheless, Lee et al. [155] found that a short term (14 days) nose-only exposure of mice to 20 nm AgNPs at concentration of 1.91 × 10^7^ particles/cm^3^ led to alterations in brain gene expression. Sub-chronic (90 days) inhalation studies showed mild, dose-dependent pulmonary inflammation and alterations in pulmonary function in rats exposed to 18 nm AgNPs [153]. In addition, inhaled AgNPs may also enter systemic circulation to become distributed to extra-pulmonary organs such as the liver and the brain, as demonstrated in studies that used ~15 nm NPs at concentrations of 1–3 × 10^6^ particles/cm^3^ [151,156]. A 90 days, an inhalation study by Sung et al. showed alterations in lung function and inflammatory responses in rats exposed to 18 nm AgNPs [153]. Additionally, the accumulation of Ag in the lungs and the liver were more evident in rats after 90 days of inhalation [103]. Silver accumulation has been also observed in the brain, the olfactory bulb, the kidney and the spleen [103,157,158]. Moreover, AgNPs (18–20 nm) were also shown to reach and cross mouse placenta in an inhalation study, where pregnant females were exposed to freshly produced aerosols for either 1 or 4 h/day during the first 15 days of gestation at a particle number concentration of 3.80 × 10^7^ part/cm^3^ [159].

In several cases, a gender-dependent difference for AgNPs accumulation in kidneys has been reported, with females exhibiting a higher concentration than males [101,153,167]. One possible explanation for the sex differences in the distribution of Ag may be hormonal regulation in the rat kidney [36]. A gender-dependent difference was also reported in terms of the persistence of pulmonary inflammation and a decrease in lung function in male rats following the termination of exposure, while females showed a gradual improvement in lung inflammation following the cessation of exposure [104]. However, the exact mechanism of sex-related differences is still not clear.

The potential mechanisms of the cardiovascular effects of lung-deposited particles were previously discussed by Nemmar et al. [168]. In this context, Holland et al. [164] investigated the effect of 20 nm AgNPs on cardiovascular injury and showed the exacerbation of cardiac ischemic-reperfusion injury following a single i.t. instillation in rats. The authors further evaluated the impact of the size (20 and 110 nm) and coating (PVP and CT) of AgNPs, and they demonstrated that the acute effect was size- and coating-independent, whereas the persistence of injury was greater for 110 nm PVP-AgNPs [165]. A significant dose-dependent effect of pulmonary-exposed PVP- and CT-AgNPs on cardiovascular homeostasis was also demonstrated in our recent study [166]. The mechanism through which lung injury occurs and how the physicochemical properties of inhaled AgNPs affect their interactions with the lung have recently begun to be investigated in vivo [100,158,162,163]. Along with in vitro studies, in vivo results have suggested that size, coating and dose affect pulmonary inflammation and cellular toxicity.

As mentioned above, besides respiratory exposure, consumer exposure to AgNPs via ingestion can also occur due to the incorporation of AgNP into products such as food containers and dietary supplements. Table 3 summarizes the important toxicity and biodistribution studies of AgNPs in rodents via oral exposure. The deposition of orally-exposed AgNPs in the GIT has been widely demonstrated in previous studies [36,169]. Jeong et al. [170] showed an increase of goblet cells in the intestine, together with a high mucus granule release in mice orally-treated with AgNPs (60 nm) at a concentration of 30 mg/kg bw/day for 28 days. In addition, AgNPs (5–20 nm) that were orally administrated for 21 days in mice (20 mg/kg of body weight) disrupted epithelial cell microvilli and intestinal glands [106]. The distribution of PVP-AgNPs (14 nm) to multiple organs including the intestine, the liver, the kidney, the lung and the brain following oral administration have been reported [169]. Several other investigators also showed that oral exposure to AgNPs may lead to liver, intestinal and neuronal damage [171,172,173]. Cases of argyria (a condition characterized by an irreversible gray or bluish gray pigmentation of the skin), irreversible neurologic toxicity, and death have been reported upon the long-term ingestion of colloidal silver [174]. The liver appears to be a major accumulation site of circulatory AgNPs [175]. In fact, PVP-AgNPs (20–30 nm) have been shown to increase oxidative stress, enhance autophagy, and deplete insulin signaling pathways following oral exposure for 90 days in the liver of male rats [173]. Changes in blood parameters indicated by a significant elevation of ALT, AST, and hepatoxicity, shown by histological damages (necrosis, hepatocytic inflammation, and the resultant aggregation of lymphocytes in liver tissue) were also observed in a study that evaluated the toxic effect of 14 days of oral exposure to AgNPs (40 nm) at doses 20 and 50 ppm in BALB/C mice [176]. Moreover, Tiwari et al. [177] determined the effect of 60 days AgNPs (10–40 nm) treatment on the kidneys of female Wistar rats at doses of 50 and 200 ppm, and they demonstrated significant mitochondrial damage, increased levels of serum creatinine, and early toxicity markers such as KIM-1, clusterin and osteopontin.

The use of AgNPs in wound dressings and other applications designed to regulate skin microbiome composition is an established strategy that has been shown to inhibit a broad range of bacteria, including *Pseudomonas aeruginosa*, *Escherichia coli* and *Staphylococcus aureus* [57,81]. Numerous reports have indicated that AgNPs promote wound healing by decreasing the inflammatory response [185,186]. Nevertheless, an aspect that remains sparsely researched is the possibility of the sensitization of the skin by these NPs. It is well known that patients with wounds are at particular risk of developing either an allergic or irritant contact dermatitis, and silver compounds are widely used in wound care. However, so far, there have been very few confirmed cases of contact dermatitis secondary to silver-containing wound products like silver sulfadiazine and skin markers that contain silver nitrate [187,188]. Contrary, there is considerable evidence for the significant transdermal penetration of AgNPs into capillaries during the use of surgical dressings, textiles, and cosmetics [189,190]. In order to elucidate the mechanism of cytotoxicity, Samberg et al. [129] evaluated the potential ability of AgNPs to penetrate porcine skin and showed the existence of the focal inflammation and localization of AgNPs on the surface and in the upper stratum corneum layers of porcine skin. Acute and sub-acute dermal studies conducted by Korani et al. [6] suggested a correlation between dermal exposure, tissue accumulation of AgNPs (100 nm), and dose-dependent histopathological abnormalities in the skin, all of which was exhibited by a reduced thickness of the papillary layer and the epidermis. Compared to animals treated with a single dose, animals that were subjected to sub-chronic exposure showed a considerable accumulation of AgNPs, as well as a dose-dependent toxic response in several organs, including the spleen, liver and skin [191].

Another potential route of AgNPs entry in the case of biomedical applications includes parenteral administration. A summary of important toxicity and biodistribution studies of AgNP in rodents via exposure of i.v. injection is given in Table 4. In a recent study, comparing the biodistribution and toxicological examinations after repeated i.v. administration of AgNPs and AuNPs in mice showed a higher deposition of AgNPs in the heart, the lung, and the kidney than that of AuNPs [175]. Moreover, the AgNPs induced adverse effects in a dose-dependent manner (the concentrations tested were 4, 10, 20, and 40 mg/kg) [175]. Another study, following the subcutaneous administration of AgNPs of different sizes in rats also revealed that the particles translocated to the blood circulation and were distributed throughout the main organs, especially in the kidney, the liver, the spleen, the brain and the lung [131]. The results also suggested the potential of AgNPs to cross the blood–brain barrier and to induce astrocyte swelling and neuronal degeneration [131]. A few studies have reported the transfer of AgNPs across the placenta in rats and mice [192,193]. Following the i.v. administration of 10 nm AgNPs at a dose of 66 µg Ag/mouse to pregnant animals on gestational days 7, 8 and 9, Ag accumulation was revealed in all examined organs, with the highest accumulation being in the maternal liver, spleen and visceral yolk sac and the lowest concentrations being in the embryos [192]. Another study comparing administration methods (i.v. versus i.p.) showed a similar localization of Ag in the liver and the spleen for both methods [193]. However, Ag was more quickly excreted from the body with i.v. administration, as compared to i.p. administration. The latter study also showed that the AgNPs could cross the placental and the blood–testes barriers, thus resulting in an accumulation in the fetus and the testes, respectively [193].

## 5. Knowledge Gaps in Human and Environmental Risk Assessment

For all NPs studies, a crucial issue remains the composition, particle surface area, surface chemistry, and the careful, accurate characterization of particle size and morphologic features, especially in the physiological environment [202]. Moreover, equally important to the latter is the control of assays and assay conditions [202]. It is only with the complete characterization of NPs and the appropriate control of assays that the results of reported studies can be comparable with those of other studies conducted with similar NMs [68,203]. Unfortunately, the characterization of materials, especially following in vivo applications, is still inadequate for many published studies. In this regard, though several characterizing tools have been developed, each has its own limitations. For instance, DLS is the most commonly used tool, especially in studies that adopt limited characterization steps, due to its accessibility, low cost, and easy handling [204]. However, its disadvantages include low resolution, multiple light scattering, sedimentation, a lack of selectivity, and a relatively low signal strength, particularly in complex biological media such as in plasma [204]. Likewise, zeta potential is affected not only by the properties of NPs but also by the nature of the solution, such as pH and ionic strength [204]. Moreover, the understanding of operating principles, as well as dealings with critical issues like sample preparation and data interpretation, proposes challenges to the application of these characterization techniques. As the outcome of particle effects is largely governed by the NP’s physicochemical properties, the thorough characterization of AgNPs is extremely important, especially when investigating in vivo effects following various routes of contact.

Toxicokinetic studies of NPs including absorption, distribution, metabolism and elimination have provided important data related to their *in vivo* behavior and risk assessment. In this context, there are very limited data on AgNPs’ toxicokinetic properties, particularly metabolism and clearance. Though several of the studies discussed in this review have demonstrated organ distribution following the translocation of AgNPs, it is not yet clear whether the distribution and effects observed were due to their particulate forms, ionic forms, or a combination of both forms. In order to find out whether the distribution of AgNPs were in ionic or particulate forms, Lee JH et al. [205] recently investigated the toxicokinetic of i.v.-administered AgNPs (10 nm) and AuNPs (14 nm), either separately or in combination, and evaluated NP clearance after a four-week recovery period. Interestingly, their data revealed that the co-administration of AgNPs with AuNPs of a similar size distribution not only decreased NPs’ distribution to organs, thus indicating a competitive cellular uptake, but also confirmed the particulate form of NP tissue distribution rather than ionic form [205]. Another potential form suggested is the distribution in secondary particle form, because NPs can interact with proteins like thiol after dissolution in ionic form. The latter was demonstrated by Liu et al. [206], who suggested that the newly formed secondary AgNPs circulated in systemic circulation and photoreduced to metallic silver, eventually contributing to agyria silver deposits in light-affected skin areas. Another study that used silver nitrates or AgNPs also demonstrated the formation of similar secondary AgNPs [106]. This in fact gives another dimension to research, as the detected AgNPs in tissues could not only be the product of exposed nanotechnology but could also have been due to any chemical forms of silver exposure that eventually transformed into secondary AgNPs. A parallel controversy pertains to the tissue clearance of accumulated silver. In spite of studies that have evaluated silver clearance with different exposure routes, exposure periods, and recovery periods, there not yet a clear understanding of whether the ionic or particulate form is eliminated from tissues.

Another aspect that has not been studied much is the impeding effects of AgNPs on susceptible populations like pulmonary disorders, obesity, hypertension, and diabetes. It is well established that the impact of air pollution is aggravated in patients with pre-existing cardiorespiratory diseases, such as asthma, and chronic respiratory diseases, such as chronic obstructive pulmonary disease, pneumonia, cystic fibrosis, and ischemic heart diseases [207]. A recent study also reported the exacerbation of autoimmune diseases to short term exposure to particulate matter (PM_10_ and PM_2.5_) [208]. However, information in regard to the effect of AgNP exposure on susceptible populations is very much limited. The pathophysiological effects of the latter could be well studied by using animal models of increased susceptibility, e.g., hypertension and diabetes, changes in blood biochemistry, acute phase response, and hepatic pathology. In this context, Ramirez-Lee et al. [209] recently evaluated the cardiovascular effect of 15 nm AgNPs by using isolated perfused hearts from male, spontaneously hypertensive rats. The authors concluded that hypertension intensified AgNP-induced cardiotoxicity due to an observed reduction in NO and an increase in oxidative stress, leading to increased vasoconstriction and myocardial contractility. Jia et al. [210] studied the effect of orally-exposed PVP-AgNPs (30 nm) in overweight mice and showed the progression of fatty liver disease from steatosis to steatohepatitis. The mechanisms proposed in the latter study were the activation of Kupffer cells, the enhancement of hepatic inflammation, and the suppression of fatty acid oxidation. Kermanizadeh, A. et al. [211] investigated AgNP-induced hepatic pathology in models representative of pre-existing alcoholic liver disease. Their data showed that following oral exposure, AgNP-induced hepatic effects were aggravated in the alcohol-pretreated mice in comparison to controls with regards to an organ-specific inflammatory response.

The evaluation of trans-generational impact is also an important point to understand the long-term effect of NPs on human health and the environment. In this context, Hartmann et al. [212], assessed the impact of pristine and waste water-borne AgNPs on the aquatic invertebrate *Daphnia magna* in a multi generation approach that covered six generations. The authors showed that while pristine AgNPs caused a significant reduction in the mean number of offspring compared with the control, the waste water-borne AgNPs had no effects on reproduction in any generation. Raj et al. [213] investigated impact of ingested AgNPs (20–100 nm) on the adult and larval stages of *Drosophila*. Their results demonstrated a significant reduction of survival, longevity, ovary size, and egg laying capability in flies fed with AgNPs compared with a control [213]. The latter effects persisted in the next generation without AgNP feeding, thereby suggesting the transgenerational effects of AgNPs. Despite these findings, the in vivo transgenerational effects of AgNPs involving higher mammalian systems or humans still remains the least explored area of NP research.

Another aspect that lacks detailed research is the effect of AgNPs on humans via various routes of exposure. In this regard, very few studies have attempted to investigate whether AgNPs can penetrate physically and functionally intact human skin [99,214]. George et al. [99] demonstrated the in vivo penetration of AgNPs (10–40 nm) by using healthy human participants with normal skin. Their data suggested that AgNPs, applied as nanocrystalline silver dressing for four-to-six days, can penetrate beyond the stratum corneum and reach as deep as the reticular dermis. A controlled, cross over time exposure (three, seven, and 14 days) study of orally dosed (10 ppm) commercial AgNPs (5–10 nm) demonstrated the absence of any changes in human metabolic, hematologic, urine, and physical findings or imaging morphology [215]. However, AgNP toxicology research with respect to susceptible individual and human exposure thus far remains understudied, and these areas are particularly important with regard to NP risk assessment.

The increasing concern about the possible impact of AgNPs on the environment and, subsequently, human health has directed researchers to focus on the in vitro and in vivo toxicity induced by these particles.

## 6. Conclusions and Recommendations for Future Studies

This paper critically reviewed and structurally presented the toxicity and biodistribution studies of AgNPs following various routes of exposure. Our conclusions drawn from these studies are listed below:

- The cytotoxic effects of AgNPs, documented in in vitro studies in various cell lines, are governed by factors such as size, shape, coating, dose and cell type.
- Toxicity and biodistribution studies, in vivo, following various routes of exposure, like inhalation, instillation, oral, dermal and intravenous, have established Ag translocation, accumulation, and toxicity to various organs.
- Both the local and distant organ effects are influenced by particle size, coating, route and duration of exposure, doses, and end point measurement time.
- There is lack of adequate and standard characterization techniques that could be adapted for studies that evaluate the toxicity of AgNPs in order to make the results of one study comparable to another by using similar NPs.
- The mechanisms of action of AgNPs are still not well understood, and there is lack of information on the potential effects of AgNP exposure on animal models of enhanced susceptibility, such as hypertension, diabetes, and asthma.

Owing to the evidence provided in this review, there are still gaps in the risk assessment of the Ag in the form of NPs both for humans and the environment. For example, it is still not clear to what extent the intact AgNPs themselves can enter the human body, whether the AgNPs undergo changes in the physiological environment, if the Ag^+^ ions released from the NPs absorbed, or if the effect observed is due to tge AgNP-induced inflammatory response or due to the ions released or due to the nanoparticulate from itself. Since no clear pathway has been proven to be the most important mechanism of AgNP-induced pathophysiological effects, we have some recommendation for future research listed below.

- In order to overcome the limitation of a single method of particle characterization and to efficiently evaluate the functional effect of synthesized particles, the characterization of AgNPs should be done by using multiple relevant techniques.
- AgNPs’ characteristics should be evaluated in an appropriate medium because interactions with a biological fluid can alter NPs’ properties, intake, and cellular effects.
- There is a need for extensive data on the biodistribution and accumulation of AgNPs, and these data should take AgNPs’ various physicochemical properties into consideration in order to get a concrete idea on the local and distant tissue toxicity of AgNPs, as well as the mechanisms behind the toxicity.
- Appropriate techniques and methodologies have to be constructed in order to estimate Ag^+^ ions originating from AgNPs in vivo and to calculate AgNPs’ surface ionization fraction in various tissues.
- AgNPs effect on animal models with pre-existing diseases like asthma, obesity, hypertension, and diabetes needs to be carried out, as toxicological consequences might be aggravated in animal models of enhanced susceptibility.
- Multi-generation studies assessing the transgenerational impact of AgNPs in higher mammalian systems needs to be carried out in order to identify the potential long-term effects of AgNPs in a more realistic scenario.
- Multidisciplinary investigations taking in account long term exposure, variable routes of exposure, and the dosing of AgNPs should be conducted in humans in order to ascertain the human toxicity threshold.

## Figures and Tables

**Table 1:** Toxicity and distribution of AgNPs following pulmonary exposure in rodents via inhalation.

Size	Dose	Model	End-Point Measurement	Effect	Tissue Accumulation	References
11–14 nm	1.73 × 10^4^/cm^3^ (low dose), 1.27 × 10^5^/cm^3^ (middle dose), (1.32 × 10^6^ particles/cm^3^ (high dose)	Sprague–Dawley rats	Inhalation 6 h/day, 5 days/week, for 4 weeks, sacrificed 1 day post last exposure	AgNPs concentration below the American Conference of Governmental Industrial Hygienists silver dust limit (100 µg/m^3^ did not produce significant toxic effects.	Lu, Li, Br, Ob	[151]
18 nm	0.7 × 10^6^ particles/cm^3^ (low dose), 1.4 × 10^6^ particles /cm^3^ (middle dose), and 2.9 × 10^6^ particles/cm^3^ (high dose)	Sprague–Dawley rats	Inhalation 6 h/day, 5 days/week, for 90 days, sacrificed 1 day post last exposure	Subchronic exposure to AgNPs compromised the lung function.	N/A	[153]
18 nm	0.6 × 10^6^ particle/cm^3^, 49 μg/m^3^(low dose), 1.4 × 10^6^ particle/cm^3^, 133 μg/m^3^ (middle dose) and 3.0 × 10^6^ particle/cm^3^, 515 μg/m^3^ (high dose)	Sprague–Dawley rats	Inhalation 6 h/day, 5 days/week, for 90 days, sacrificed 1 day post last exposure	Silver accumulation in kidney was gender-dependent. Dose-dependent increase of bile duct hyperplasia in AgNP-exposed liver.	Lu, Li, Br, Ob, Ki	[103]
10 nm (PVP-coated)	3.3 ± 0.5 mg/m^3^ or 31 µg/g lung	Male C57Bl/6 mice	Inhalation 4h/day, 5 days a week, for 10 days, sacrifice at 1 hr and 21 days post last exposure	Subacute inhalation of nanosilver induced minimal pulmonary toxicity.	Lu	[152]
15 nm; 410 nm	179 μg/m^3^ and 167 μg/m^3^ or 7.9 × 10^6^ particles/mm^3^ and 118 particles/mm^3^ for 15 and 410, respectively	Male Fischer rats	Inhalation 6 h/day, 4 consecutive days, sacrifice at 1 and 7 days post exposure	Size-dependent effect on pulmonary toxicity after inhalation of similar mass concentration of 15 and 410 nm AgNPs.	Lu, Li	[158]
15 nm	8, 28 µg	BrownNorway and Sprague–Dawley rats	Inhalation 3 h/1 day and 3 h/4 days. Sacrifice at 1 and 7 days post last exposure	AgNPs induced an acute pulmonary neutrophilic inflammation with the production of proinflammatory and pro-neutrophilic cytokines.	Lu	[160]
20 nm; 110 nm (CT-coated)	7.2 ± 0.8 mg/m^3^ and 5.3 ± 1.0 mg/m^3^ or 86 and 53 µg/rat for C20 and C110, respectively	Male Sprague–Dawley rats	Inhalation 6 h/1 day, sacrifice at 1, 7, 21, and 56 days postexposure	Delayed peak and short-lived inflammatory and cytotoxic effects in lungs with greater response due to smaller sized nanoparticles.	N/A	[161]
18–20 nm	3.80 × 107 part. /cm^−3^	Female C57BL/6 mice	Inhalation for 1 h/day or 4 h/day, for first 15 days of gestation, sacrificed 4 h post last exposure	Increased number of resorbed fetuses associated with reduced estrogen plasma levels, in the 4 h/day exposed mothers.	Lu, Sp, Li, Pl	[159]

Abbreviations: lung (Lu), liver (Li), spleen (Sp), kidney (Ki), olfactory bulb (Ob), brain (Br), placenta (Pl), polyvinylpyrrolidone (PVP), and citrate (CT).

**Table 2:** Toxicity and distribution of AgNPs following pulmonary exposure in rodents via intratracheal instillation.

Size	Dose	Model	End-Point Measurement	Effect	Tissue Accumulation	References
20 nm; 110 nm (PVP- and CT-coated)	0.5, 1 mg/kg	Male Sprague–Dawley rats	Single i.t. instillation, sacrifice at 1, 7 and 21 days post exposure	Coating- and size-dependent AgNPs retention in lungs. PVP-coated AgNPs had less retention over time and larger particles were more rapidly cleared from large airways than smaller particles.	Lu	[162]
20 nm; 110 nm (PVP- and CT-coated)	0.1, 0.5, 1 mg/kg	Male Sprague–Dawley rats	Single i.t. instillation, sacrifice at 1, 7 and 21 days post exposure	Smaller sized AgNPs produced more inflammatory and cytotoxic response. Larger particles produce lasting effects post 21 days instillation.	N/A	[163]
20 nm (CT-capped)	1 mg/kg	Male Sprague–Dawley rats	Single i.t. instillation, sacrifice at 1 and 7 days post exposure	AgNP resulted in exacerbation of cardiac ischemic-reperfusion injury.	N/A	[164]
20 nm; 110 nm	1 mg/kg	Male Sprague–Dawley rats	Single i.t. instillation, sacrifice at 1 and 7 days post exposure	Both sizes of AgNP resulted in exacerbation cardiac I/R injury 1 day following instillation independent of capping agent. Persistence of injury was greater for 110 nm PVP-capped AgNP following 7 days instillation.	N/A	[165]
50 nm; 200 nm (PVP-coated)	0.1875, 0.375, 0.75, 1.5, 3 mg/kg	Female Wistar rats	Single i.t. instillation, sacrifice at 3 and 21 days post exposure	Focal accumulation of Ag in peripheral organs along with transient inflammation in lung.	Li, Sp, Ki	[157]
50 nm; 200 nm (PVP- and CT-coated)	0.05, 0.5, 2.5 mg/kg	Female BALB/C mice	Single i.t. instillation, sacrifice 1 day post instillation	Size-, dose- and coating-dependent pro-inflammatory effects in healthy and sensitized lungs following pulmonary exposure to AgNPs.	N/A	[100]
10 nm	0.05, 0.5, 5 mg/kg	BALB/C mice	Single i.t. instillation, sacrifice at 1 and 7 days post exposure	Oxidative stress, DNA damage, apoptosis in heart. Induced prothrombotic events and altered coagulation markers.	N/A	[166]

Abbreviations: lung (Lu), liver (Li), spleen (Sp), kidney (Ki), intratracheal (i.t.), polyvinylpyrrolidone (PVP), and citrate (CT).

**Table 3:** Toxicity and biodistribution of AgNPs in rodents via oral exposure.

Size	Dose mg/kg	Model	End-Point Measurement	Effect	Tissue Accumulation	References
56 nm	30, 125, 500	F344 rats	Daily exposure for 90 days, sacrifice 24 h post last exposure	Accumulation of silver in kidneys was gender-dependent, with a 2-fold increase in female kidneys. Liver is the target of silver toxicity for both male and female rats.	Li, Ki, Br, Lu, Bl	[102]
15 nm; 20 nm	90	Male Sprague Dawley rats	Daily exposure 28 days, sacrifice 24 h, 1 week, 8 weeks post last exposure	Main target organ for AgNPs and AgNO_3_ upon oral exposure are the liver and spleen. Silver was cleared from all organs after 8 weeks post dosing except brain and testes.	Li, Sp, Te, Ki, Br, Lu, Bl, Blr, Ht	[106]
20 nm	820	Male Sprague Dawley rats	Daily exposure for 81 days, sacrifice 24 h post last exposure	AgNPs induces liver and cardiac oxidative stress and mild inflammatory response in liver.	N/A	[178]
20, nm; 110 nm (PVP- and CT-coated)	0.1, 1, 10	Male C57BL/6NCrl mice	3 days exposure, sacrifice at 1 and 7 days post exposure	Acutely ingested AgNP, irrespective of size or coating, are well-tolerated in rodents even in markedly high doses and associated with predominant fecal accumulation.	Absence of tissue accumulation	[179]
10 nm; 75 nm; 110 nm	9, 18, 36	Sprague Dawley rats	Daily exposure for 13 weeks. Sacrifice 24 h post last exposure	Silver accumulation in tissues showed a size-dependent relationship 10>75>110. Statistically significant difference in distribution and accumulation of silver in male and female rats. No toxic effect on blood, reproductive and genetic system tested was observed.	Ki, Sp, Li, Ht, Ut	[36]
20–30 nm (PVP-coated)	50, 100, 200	Male Sprague Dawley rats	Daily exposure for 90 days, sacrifice 24 h post last exposure	Though AgNPs accumulated in hepatic and ileum cells, no harmful effects in liver and kidney, as well as no histopathological, hematological and biochemical markers changes was observed.	Il, Li, Ki, Br, Th, Spl	[180]
20–30 nm (PVP-coated)	50, 100, 200	Male Sprague Dawley rats	Daily exposure for 90 days, sacrifice 24 h post last exposure	High dose showed an increase of sperm morphology abnormalities.	N/A	[181]
10 ± 4 nm (CT-capped)	0.2	Male Wistar rats	Daily exposure for 14 days, sacrifice 24 h post exposure	Prolonged low dose AgNPs exposure induced oxidative stress in brain but not in liver.	N/A	[182]
91.71 ± 1.6 nm	0.5	Male Wistar rats	Daily exposure for 45 days, sacrificed 24 h post exposure	AgNPs caused significant oxidative stress compared to TiO_2_NP with similar dose concentration.	Bl, Li, Ki	[183]
20 nm; 110 nm	10	Pregnant Sprague Dawley rats	Single exposure, sacrificed at 24 h and 48 h post exposure	Silver enters and crosses placenta regardless of route and form of silver.	Ce, LI, Pl, Ki, Bl	[184]
20–30 nm (PVP-coated)	50, 100, 200	Male Sprague Dawley rats	Daily exposure for 90 days, sacrifice 24 h post last exposure	High dose of AgNPs induced hepatocellular damage by increased ROS production, enhanced autophagy and depleted insulin signaling pathway.	N/A	[173]

Abbreviations: lung (Lu), liver (Li), spleen (Sp), kidney (Ki), brain (Br), heart (Ht), bladder (Bl), uterus (Ut), thymus (Th), cecum (Ce), placenta (Pl), polyvinylpyrrolidone (PVP), and citrate (CT).

**Table 4:** Toxicity and biodistribution of AgNPs in rodents via exposure through intravenous injection.

Size	Dose	Model	End-Point Measurement	Effect	Tissue Accumulation	References
15–40 nm	4, 10, 20, 40 mg/kg	Male Wistar rats	32 days i.v, sacrifice 24 h post last i.v administration	AgNPs in doses <10mg/kg is safe, while >20mg/kg is toxic.	Li, Ki	[194]
21.8 nm	7.5, 30, 120 mg/kg	ICR mice	Single i.v, parameters measured at 1,7,14 days post injection	Inflammatory reactions in lung and liver cells were induced in mice treated at the high dose of AgNPs. Gender-related differences in distribution and elimination of AgNPs, elimination in female is longer than the males.	Sp, Li, Lu, Ki	[195]
20 nm; 200 nm	5 mg/kg	Male Wistar rats	Single i.v, sacrifice at 1, 7, 28 days post i.v administration	High tissue concentration of silver in tissues of 20 nm group as compared to 200 nm groups	Li, Sp, Ki, Lu, Br, Ur, Fe	[196]
7.2 ± 3.3 nm	5, 10, 45 mg/kg	Male Sprague–Dawley rats	Daily tail vein injection for 3 consecutive days, parameters measured at 1 and 3rd day	Decrease in body weight and locomotor activity.	N/A	[197]
20, nm; 100 nm	0.0082, 0.0025, 0.074, 0.22, 0.67, 2, 6 mg/kg	Wistar rats	28 days, repeated i.v, sacrifice at 24 h post last injection	Immune system is the most sensitive parameter affected by AgNPs; reduced thymus weight, increased spleen weight and spleen cell number, strongly reduced NK cell activity, and reduced IFN-γ production were observed.	N/A	[37]
20 nm	0.0082, 0.0025, 0.074, 0.22, 0.67, 2, 6 mg/kg	Male Wistar rats	28 days, repeated i.v, sacrifice at 24 h post last injection	AgNPs suppress the functional immune system.	N/A	[35]
10 nm (CT-coated)	1 mg/kg	CD1 mice	IV administration, once every 3 days, sacrifice at 15, 60 120 days post initial exposure	AgNPs induced toxicity to male reproduction, altered Leydig cell function, increased testosterone level.	Te	[198]
10 nm; 75 nm; 110 nm (CT-coated)	0.1 mg/kg	Female BALB/C mice	Single dose injection sacrifice at 4h, 1, 3 or 7 days post injection. Multi dose: i.v injection on day 1, 4 and 10, sacrifice at 7 days post last injection	Injection of a single dose of AgNPs induced a less toxicity in liver and lung tissues than that induced by multi-dose injections. The toxic effect of AgNPs was time-dependent.	N/A	[199]
10 nm; 40 nm, 100 nm (PVP- or CT-coated)	10 mg/kg	Male CD-1 (ICR) mice	Single i.v, sacrifice at 24 h post injection	10 nm AgNP group showed increased silver distribution and overt hepatobiliary toxicity compared to larger ones.	Sp, Li, Lu, Ki, Bl, Br	[68]
3 ± 1.57 nm	11.4–13.3 mg/kg	Male KunMing mice	i.v. injection 2 times/week for 4 weeks, sacrificed at 1, 28, 56 days post injection	AgNPs preferentially accumulated in all major organs compared to other metallic NPs.	Li, Sp, Ki, He, Lu, Te, Fe, Bl, In, St, Br, SV	[175]
20 nm; 110 nm	1 mg/kg	Pregnant Sprague Dawley rats	Single exposure, sacrificed at 24 and 48 h post exposure	Silver crosses the placenta and is transferred to the fetus regardless of the form of silver.	Sp, Pl, Li, Lu, Ce, Bl, Ki	[184]
6.3–629 nm	5 mg/kg	Female Sprague Dawley rats	Single exposure, sacrificed at 24 h	Histopathologically, AgNPs caused mild irritation in thymus and spleen and significantly increased chromosome breakage and polyploidy cell rates.	Lu, Sp. Li, Ki, Th, Ht	[200]
20 nm; 110 nm (PVP or CT)	701.75 μg/kg	Pregnant female Sprague Dawley rats	Single i.v administration	Exposure to CT-AgNPs is associated with changes in fetal growth and increased contractile force in both uterine and aortic vessels.	N/A	[201]

Abbreviations: lung (Lu), liver (Li), spleen (Sp), kidney (Ki), brain (Br), heart (Ht), bladder (Bl), uterus (Ut), thymus (Th), cecum (Ce), placenta (Pl), intestine (It), stomach (St), feces (Fe), urine (Ur), intravenous (i.v.), polyvinylpyrrolidone (PVP), and citrate (CT).

## Abbreviations

AuNPs	Gold nanoparticles
AgNPs	Silver nanoparticles
Bl	Bladder
Br	Brain
Ce	Caecum
CNTs	Carbon nanotubes
CT	Citrate
ENMs	Engineered nanomaterials
Fe	Feces
FeO	Iron Oxide
Ht	Heart
It	Intestine
i.p.	Intra-peritoneal
i.t.	Intra-tracheal
i.v.	Intra-venous
Ki	Kidney
LDH	Lactate Dehydrogenase
Lu	Lung
Li	Liver
NM	Nanomaterials
Pl	Placenta
PVP	Polyvinylpyrrolidone
ROS	Reactive Oxygen Species
Sp	Spleen
St	Stomach
Th	Thymus
TiO_2_	Titanium Oxide
Ur	Urine
Ut	Uterus
